# Altered Semmes–Weinstein monofilament test results are associated with oxidative stress markers in type 2 diabetic subjects

**DOI:** 10.1186/s12967-017-1291-8

**Published:** 2017-09-06

**Authors:** Sergio Martinez-Hervás, Mercedes Molina Mendez, José Folgado, Carmen Tormos, Pilar Ascaso, Marta Peiró, Jose T. Real, Juan F. Ascaso

**Affiliations:** 1 Service of Endocrinology and Nutrition, Hospital Clínico Universitario de Valencia, Avda Blasco Ibañez, 17, 46010 Valencia, Spain; 2 CIBER de Diabetes y Enfermedades Metabólicas asociadas (CIBERDEM), Barcelona, Spain; 3 Department of Medicine, University of Valencia and INCLIVA, Valencia, Spain; 4 Department of Biochemistry and Molecular Biology, University of Valencia, Valencia, Spain; 5 CIBER de Obesidad, Madrid, Spain

**Keywords:** Glutathione system, Malondialdehyde, Oxidative stress, Semmes–Weinstein monofilament test, Peripheral polyneuropathy, Type 2 diabetes mellitus

## Abstract

**Background:**

Different lines of evidence suggest that oxidative stress (OS) is implicated in the pathogenesis of diabetic neuropathy. The Semmes–Weinstein monofilament (SWM) test is an efficient tool for evaluating diabetic polyneuropathy and diabetic foot. In this study, we analyzed the association between OS markers and altered SWM test results in type 2 diabetes (T2DM) patients.

**Methods:**

Seventy T2DM patients were studied and 34 showed altered SWM results. The clinical and biochemical parameters were determined using standardized methods. Levels of oxidized glutathione (GSSG) and malondialdehyde (MDA) were measured in circulating mononuclear cells using high-performance liquid chromatography.

**Results:**

We found that T2DM patients with altered SWM test results had significantly higher GSSG (3.53 ± 0.31 vs. 3.31 ± 0.35 mmol/ml, *p* < 0.05) and MDA (1.88 ± 0.16 vs. 1.75 ± 0.19 nmol/ml, *p* < 0.01) values compared to diabetic patients with normal SWM test outcomes. Moreover, altered SWM test results were independently related to age, glycosylated hemoglobin, and GSSG levels, but there was no association between OS markers and altered neuropathy sensitivity score (NSS) values.

**Conclusions:**

Alteration of the glutathione system and MDA values in T2DM patients are associated with loss of proprioceptive (pressure) sensitivity, but not with symptomatic polyneuropathy (as evaluated by NSS). This finding may be important for understanding how OS affects distal symmetric polyneuropathy in diabetic patients.

## Background

The Semmes–Weinstein monofilament (SWM) test is an efficient tool for evaluating diabetic polyneuropathy and diabetic foot. The SWM test can be used to diagnose the absence of proprioceptive sensitivity (i.e. individuals’ ability to detect pressure) and to indicate the presence of distal nerve lesions in type 2 diabetes (T2DM) patients. Thus, the SWM test is use as part of a semi-quantitative test panel to diagnose distal symmetric polyneuropathy [1]. In addition, a pathological SWM test result indicates a high risk for foot ulcers and amputation. Foot ulceration is a complication of DSPN and is a significant cause of morbidity and mortality in diabetic patients [2].

The etiology of diabetic DSPN seems to be heterogeneous, but previous data strongly suggests that the increased production of reactive oxygen species (ROS) induced by hyperglycemia plays a major role in the development of this complication [3]. Moreover, several lines of evidence suggest that oxidative stress (OS) is implicated in the pathogenesis of diabetic neuropathy [3, 4].

Experimental studies have demonstrated that increased intracellular advanced glycosylation end product (AGE) formation leads to ROS-induced DNA damage [5, 6]. In addition, increased OS is detected in peripheral and dorsal root ganglion nerves and vascular nerve endothelial cells in presence of hyperglycemia [7–9]. Moreover, the presence of decreased reduced glutathione (GSH) levels and increased oxidized lipoproteins is associated with nerve damage, mitochondria vacuolization, and neuronal apoptosis [10]. This pathological state induces a reduction in nerve conduction, alters neurotrophy, and promotes neuronal apoptosis: all typical findings in DSPN [11].

In clinical studies, an increase in peroxynitrite and oxidated lipoprotein levels has been found in diabetic patients with polyneuropathy compared to diabetic subjects without polyneuropathy, thus supporting the role of OS in DSPN [12]. Moreover, a decreased total antioxidant status has been reported in T2DM patients with DSPN [12, 13]. We recently observed an association between OS markers and the presence of DSPN in T2DM patients [14]. Thus, the aim of this study was to analyze the association between malondialdehyde (MDA) and GSH/oxidized glutathione (GSSG) ratio as markers of OS and an altered SWM test in T2DM patients. We hypothesized that increased consumption of relevant intracellular antioxidant factors (such as GSH) or an increase in OS markers (GSSG and MDA) would be associated with altered SWM test results in T2DM patients.

## Methods

### Subjects

We studied 70 non-related T2DM patients, selected by consecutive sampling at the Diabetic Unit of our center and at two other primary health care centers. All the subjects were from the metropolitan area of Valencia, Spain. Diabetes was diagnosed using ADA guidelines recommendations [15].

T2DM patients were divided into two groups according to the presence of altered SWM test results or not.

The inclusion criteria were as follows: adult patients (aged ≥40 years) with type 2 diabetes. Exclusion criteria were: the presence of type 1 diabetes, heart failure [defined as New York Heart Association (NYHA) class III or IV classifications], renal disease (creatinine clearance <30 ml/min/1.72 m^2^), cirrhosis, vitamin supplementation, vitamin B12 or folate deficiency, hypothyroidism, systemic illness, cancer, smoking habit, consumption of >30 g alcohol/day, or any other disease, condition, or medication use known to cause neuropathy. Patients with Charcot arthropathy, foot ulcers, and/or bilateral amputation were also excluded. All subjects continued using their normal hypoglycemic treatment and medication for other cardiovascular risk factors.

For comparison with T2DM subjects, we have studied 55 healthy volunteers randomly recruited among plasma donors and investigators of our centre. The healthy volunteers were non-obese (BMI < 30 kg/m^2^), normolipidemic (total cholesterol <200 mg/dl and triglycerides <150 mg/dl) and normoglycemic (fasting plasma glucose <110 mg/dl) subjects. They also have not a personal or family history of dyslipidemia, cardiovascular disease or diabetes.

The ethics committee at our hospital approved the study and all the patients gave their written informed consent to participation in the study.

### Clinical and anthropometric parameters

A medical history and physical examination was carried out on all the patients, according to a research protocol. In a sitting position, the patient’s blood pressure was measured with a mercury sphygmomanometer after a 10-min rest period; their body mass index (BMI) was calculated as their weight in kilograms divided by their height in meters squared, and abdominal circumference was measured in centimeters at the point between the lower costal rim and the iliac crest. The same investigator took all the measurements.

Sensory neuropathy (loss of sensation) in the lower extremities was determined through the SWM test, by applying 10 g of force with a 5.07-guage monofilament. Three plantar sites on each foot were explored: the hallux (big toe) and base of the first and fifth metatarsals. The monofilament was not applied to areas with calluses or other structural abnormalities. A score of 1 was given for each site explored where the patient perceived the force applied in at least 2 of 3 attempts; a score of 0 was given if there was lack of perception at the site. The score was considered abnormal if the sum of all scores for both feet was between 0 and 4, while scores of 5 or 6 were considered normal [16]. The same experienced physician performed all the procedures for every participant.

The neuropathy sensitivity score (NSS) was used to quantify the presence and severity of symptomatic DSPN [17]: a NSS score of 3 or more was considered abnormal. Peripheral vascular disease (PVD) was assessed through the ankle brachial index (ABI) test, i.e. the ratio of systolic blood pressure in the ankle to that in the brachial artery. ABI was measured in the supine position using a hand-held continuous-wave Doppler ultrasound (bi-directional Smartdrop™ 20). PVD was defined by an ABI score <0.9 in either leg [18]. Scores above 1.2 were excluded from the statistical analysis due to possible arterial stiffness and because of the impossibility of accurately assessing the grade of PVD.

### Biochemical parameters

Blood samples were drawn following an overnight 12 h fasting period. Fasting glucose, total cholesterol, triglycerides, and high-density lipoprotein cholesterol were measured using standard methods based on enzymatic determination [19–21]. Glycosylated hemoglobin (HbA1c) was measured using a high-performance liquid chromatography (HPLC) assay [22], urinary albumin concentration was determined by a standard radioimmunoassay, and the estimating glomerular filtration rate (GFR) was calculated by using the modification of diet in renal disease equation (MDRD) [23].

OS markers were determined in circulating mononuclear cells (CMCs) isolated by Ficoll-Hypaque methods, as previously reported [24]. GSSG and GSH were determined using HPLC with UV detection [25] and MDA was analyzed by HPLC alone [26].

### Statistical analyses

All analyses were conducted using the Statistical Package for Social Sciences (SPSS) software (version 14, SPSS, Chicago, IL, USA). Results are expressed as the mean ± the standard deviation (*SD*). The *p*-values were two-tailed and a *p* value of less than 0.05 was considered significant. For the comparison of means, continuous variables were tested by using the t Student test. Percentages were compared using the Chi squared test. Pearson correlation was used to assess the degree of association between two quantitative variables, such as that found between GSH, GSSG, or MDA and clinical or biological parameters. We used multiple regression analysis to identify independent variables for predicting altered SWM test outcomes.

## Results

The clinical, anthropometric, and biochemical characteristics of the three study groups (healthy volunteers, T2DM patients with or without altered SWM test results) are shown in Tables 1 and 2.

**Table 1:** General, anthropometric and biochemical characteristics of type 2 diabetic patients classified according to the presence of altered SWM test

	T2DM with altered SWM test (n = 34)	T2DM without altered SWM test (n = 36)
Age (years)	68.97 ± 7.99	69.11 ± 11.65
Gender (M/F)	13/21	11/25
Smoking habit n (%)		
No	30 (88)	27 (80)
Yes	4 (12)	9 (20)
BMI (kg/m^2^)	30.61 ± 4.78	32.1 ± 5.86
Waist circumference (cm)	107.01 ± 9.86	108.84 ± 12.86
Systolic blood pressure (mmHg)	152.15 ± 20.53	147.03 ± 18.12
Diastolic blood pressure (mmHg)	80.68 ± 12.92	85.92 ± 11.96
TC (mg/dl)	192.38 ± 37.42	187.77 ± 45.29
TG (mg/dl)	155.74 ± 70.59	146.49 ± 89.49
HDLc (mg/dl)	48.88 ± 11.81	47.66 ± 11.45
Fasting glucose (mg/dl)	165.59 ± 48.16	159.49 ± 52.1
HbA1c (%)	7.98 ± 1.57	7.41 ± 1.55
Creatinine (mg/dl)	1.0 ± 0.36	0.92 ± 0.38
GFR (ml/min/1.72 m^2^)	85.6 ± 35.1	86.3 ± 56.22
Duration of diabetes (years)	13.75 ± 11.06	10.03 ± 9.38
Prevalence of coronary heart disease n (%)	8 (23)	7 (20)
Prevalence of diabetic retinopathy n (%)	13 (39)*	5 (13)
Prevalence of hypertension—n (%)	26 (76)	23 (63)
Prevalence of dyslipidemia—n (%)	24 (70)	28 (77)
ABI	0.85 ± 0.16	0.87 ± 0.17
Diabetic treatment		
Oral agents—n (%)	16 (46)	30 (84)*
Insulin—n (%)	9 (26)	2 (5)
Both—n (%)	9 (26)	4 (11)

All values are indicated as mean ± standard deviation (SD)

*T2DM* type 2 diabetic subjects, *TC* total cholesterol, *TG* triglycerides, *HDLc* high density lipoprotein cholesterol, *GFR* glomerular filtration rate (calculated by MDRA), *ABI* ankle brachial index, *SWM* Semmes–Weinstein monofilament test

* p < 0.05

**Table 2:** General, anthropometric and biochemical characteristics of type 2 diabetic patients and healthy volunteers

	Healthy volunteers (n = 55)	T2DM with altered SWM test (n = 34)	T2DM without altered SWM test (n = 36)
Age (years)	57.9 ± 4.5*	68.97 ± 7.99	69.11 ± 11.65
Gender (M/F)	24/31	13/21	11/25
BMI (kg/m^2^)	27.1 ± 4.74*	30.61 ± 4.78	32.1 ± 5.86
Waist circumference (cm)	86.1 ± 10.6*	107.01 ± 9.86	108.84 ± 12.86
Systolic blood pressure (mmHg)	114.4 ± 12.3*	152.15 ± 20.53	147.03 ± 18.12
Diastolic blood pressure (mmHg)	70.8 ± 9.7*	80.68 ± 12.92	85.92 ± 11.96
TC (mg/dl)	187.2 ± 32.5	192.38 ± 37.42	187.77 ± 45.29
TG (mg/dl)	97.3 ± 43.2*	155.74 ± 70.59	146.49 ± 89.49
Fasting glucose (mg/dl)	90.2 ± 6.5*	165.59 ± 48.16	159.49 ± 52.1

All values are indicated as mean ± standard deviation (SD)

*T2DM* type 2 diabetic subjects, *TC* total cholesterol, *TG* triglycerides, *HDLc* high density lipoprotein cholesterol, *BMI* body mass index, *SWM* Semmes–Weinstein monofilament test

* p < 0.05

Comparing the studied parameters between T2DM groups (Table 1), we observed no significant differences in age, gender distribution, disease duration, BMI, waist circumference, smoking habits, HbA1c, or renal function between the groups. In addition, the two T2DM studied groups showed similar ABI, systolic and diastolic blood pressure values, lipid profiles, and GFRs. As expected, we found significant differences in the prevalence of diabetic retinopathy in T2DM patients with altered SWM test results compared to those with normal SWM test outcomes (39% vs. 13%), and this latter group showed significantly higher consumption of oral hypoglycemic agents than the former group.

Healthy volunteers were younger, leaner and showed significantly decrease fasting tryglicerides and glycemia, as expected (Table 2).

As shown in Table 3, we found that GSSG values were significantly increased in T2DM patients with altered SWM test results compared to those without an altered SWM test result (3.53 ± 0.31 vs. 3.31 ± 0.36 mmol/ml, *p* < 0.05). Levels of MDA, a marker of lipid peroxidation, were also significantly higher in diabetic patients with altered SWM test results compared to those with normal results (1.88 ± 0.16 vs. 1.75 ± 0.19, *p* < 0.01). Altered SWM test outcomes were associated with GSSG (r = 0.328, *p* = 0.006), MDA (r = 0.328, *p* = 0.006), age (r = −0.446, *p* < 0.001), MDRD (r = 0.314, *p* = 0.009), and NSS (r = −0.285, *p* = 0.017), whereas normal SWM test results were not.

**Table 3:** Reduced glutathione (GSH), oxidized glutathione (GSSG), GSH/GSSG ratio, and MDA in healthy volunteers and diabetic subjects

	Healthy volunteers (n = 55)	T2DM with altered SWM test (n = 34)	T2DM without altered SWM test (n = 36)
GSH (nmol/ml)	22.53 ± 3.82**	19.81 ± 4.45	19.50 ± 4.36
GSSG (nmol/ml)	2.78 ± 1.28**	3.53 ± 0.31*	3.3 ± 0.35
GSH/GSSG ratio	10.1 ± 5.29**	5.67 ± 1.43	5.96 ± 1.41
MDA (nmol/ml)	1.37 ± 0.23**	1.88 ± 0.16*	1.75 ± 0.19

All values are indicated as mean ± standard deviation

*T2DM* type 2 diabetic subjects, *MDA* malonildialdehyde, *GSH* reduced glutathione, *GSSG* oxidized glutathione, *oxidized-LDL* oxidized low-density lipoprotein, *SWM* Semmes-Weinstein monofilament test

* p < 0.05 T2DM subjects with altered SWM test vs T2DM subjects without altered SWM test

** p < 0.05 healthy volunteers vs T2DM subjetcs

Healthy volunteers showed statistical significant decrease GSSG and MDA values compared to T2DM subjects (Table 3). In addition, healthy volunteers have significantly higher GSH and GSH/GSSG ratio compared to T2DM patients. These data indicate that T2DM have OS, independently of SWM test results.

Using the SWM test results as a dependent variable and age, diabetes evolution time, HbA1c, GSSG, and MDRD as independent variables, age, HbA1c, and GSSG were significant predictors of altered SWM test outcomes in our logistic regression model analysis (beta = −0.122, *p* = 0.01; 0.438, *p* = 0.039; and 2.72, *p* = 0.008, respectively).

We also classified the studied patients into two groups according to the presence or absence of a NSS score of 3 or more (presence of symptomatic polyneuropathy) (Table 4); there were no statistically significant differences in the clinical and biochemical parameters between groups, except for gender distribution. Moreover, NSS scores of 3 or more were not associated with OS markers.

**Table 4:** General characteristics, lipids, glutathione and MDA values in type 2 diabetic subjects divided according to the presence of NSS ≥ 3

	T2DM with NSS ≥ 3 (n = 23)	T2DM without altered NSS ≥ 3 (n = 47)
Age (years)	68.48 ± 8.9	61.6 ± 11.4
Gender (M/F)	12/11	12/35*
BMI (kg/m^2^)	31.68 ± 5.1	31.14 ± 5.5
ABI	0.87 ± 0.16	0.86 ± 0.16
Glucose (mg/dl)	168.26 ± 52.6	159.6 ± 48.8
TC (mg/dl)	190. 1 ± 30.95	190.04 ± 46.1
TG (mg/dl)	149.83 ± 49.1	151.65 ± 92.52
HbA1c (%)	7.62 ± 1.41	7.73 ± 1.66
GSH (nmol/ml)	20.51 ± 3.82	19.25 ± 4.6
GSSG (nmol/ml)	3.45 ± 0.34	3.41 ± 0.35
MDA (nmol/ml)	1.83 ± 0.18	1.81 ± 0.18

All values are indicated as mean ± standard deviation

*T2DM* type 2 diabetic subjects, *BMI* body mass index, *ABI* ankle brachial index, *TC* total cholesterol, *TG* triglycerides, *GSH* reduced glutathione, *GSSG* oxidized glutathione, *MDA* malonildialdehyde

* p < 0.05

## Discussion

Our results show that diabetic patients with altered SWM test results have significantly increased GSSG levels compared with diabetic patients without altered SWM test outcomes. These results suggest that patients with altered sensitivity (ability to discriminate pressure) in their lower extremities and a high risk of foot ulcers consume increased amounts of glutathione. In contrast, GSH, GSSG, and MDA values are not associated with symptomatic polyneuropathy (as evaluated by NSS) in T2DM patients. Moreover, known factors that modulate OS such as age, gender, renal function, glycemic control (HbA1C), and smoking habits were similar in both the T2DM groups we studied, highlighting the association between OS markers and altered SWM test results we outline here. In addition, multiple-factor regression analysis showed that GSSG values are a strong independent predictor of altered SWM test results in T2DM subjects.

Supporting our results, data from previous studies underline how insufficient cellular antioxidative defense mechanisms strongly contribute to nerve damage. Kasznicki et al. demonstrated that the activity of cellular antioxidant enzymes such as superoxide dismutase (SOD), catalase (CAT), and glutathione peroxidase (GPx), in peripheral blood cells, as well as the total plasma antioxidant capacity, were diminished in diabetic patients with DSPN [12].

GSH forms part of the defense against ROS, and is the most important known water-soluble antioxidant, especially in reducing excess intracellular hydrogen peroxide [27] and in a large number of cellular detoxification reactions [28]. In neurons, its synthesis depends on glia glutamylcysteine synthetase, which is very sensitive to its depletion [29, 30]. Therefore, in the presence of chronic hyperglycemia, increased ROS can induce intracellular depletion of GSH and consequently, excess OS in neurons, thus producing neuron and nerve damage.

In contrast, increased GSSG values indicate a decline in the ROS defense system [31]: El-Boghdady et al. found a significant decrease in erythrocyte GSH in diabetic patients with and without DSPN compared to healthy subjects [32]. Similar results were found in a study in T2DM patients with DSPN (as evaluated using the neuropathy disability score; NDS), revealing a decrease in GSH and the GSH/GSSG ratio in circulating monocytes in DSPN patients compared to those without DSPN [14].

We also found a significant association between MDA, a byproduct of lipid peroxidation and an OS marker, and altered SWM test results in T2DM patients. In addition, increased erythrocyte MDA levels have been found in diabetic patients [33], and different studies have shown that higher levels of MDA are related to microvascular [34] or macrovascular risk complications in diabetic patients [35]. Moreover, increased MDA levels were specifically associated with the presence of DSPN [32].

These findings are important because a decrease in antioxidant defenses is considered to contribute to oxidative damage in lipids, proteins, and DNA, resulting in neuron apoptosis [8, 36]. Different studies have demonstrated that chronic hyperglycemia leads to increased intracellular ROS production and as a consequence, increased OS in diabetic patients [3, 37]. The effect of hyperglycemia in mitochondria is partially mediated through superoxide anion overproduction [38] and an increase in the production of ROS has been associated with the development of chronic microvascular complications of diabetes [9, 10].

The before mentioned clinical studies support the idea that an increase in OS may be a key pathogenic factor in neural injury that could contribute to the development of neuropathy in diabetic patients [39]. Indeed, several important experimental animal-model studies also support this hypothesis; in diabetic rats an antioxidant reduction was discovered in the sciatic nerve [40, 41] and an increase in OS products in peripheral nerves, glial cells, and dorsal root ganglion has been associated with hyperglycemia [42–44]. Moreover, all these findings have been associated with myelopathy, mitochondrial vacuolation, and eventual neuron and glia apoptosis [8]. Moreover, a recent study proposed that carbonylated proteins, whose presence in myelin is induced by OS, contribute to the protein structural modification and aggregation observed in rodents with T2DM, the key mechanism involved in driving deficits and neurotropic deterioration. They also found morphological abnormalities in peripheral nerve myelin, similar to that observed in nerves affected by DSPN [45].

Interestingly, the increase in ROS found in glucose-intolerance states is associated with neural injury and decreased electrophysiological nerve parameters [46]. These findings support the hypothesis that nerve injury is very sensitive to moderate hyperglycemia mediated by ROS and that it is likely an early stage in the formation of neural lesions in diabetic neuropathy. On the other hand, in dorsal root ganglia neural cells cultured in vitro and exposed to hyperglycemic medium, ROS production produced mitochondrial depolarization and cell death [47]. In another study, hyperglycemia was associated with DNA methylation alterations and an increase in the presence of ROS products in Schwann cells [48]. In fact, some authors have suggested that even hyperglycemic spikes may be sufficient to generate OS and associated diabetic neuron damage [49].

Previous data from our research group [14] demonstrated an association between OS markers and the presence of DSPN in T2DM. Based on these findings, and the established relationship between OS and diabetic complications, the results we present here suggest that an increased OS status may contribute, at least in part, to nerve damage, which can be measured as altered SWM test results in T2DM patients. However, additional studies will be required to confirm and further explore these findings. Notwithstanding, this present study does have some limitations. Firstly, it is a case–control study and so it cannot establish a causal relationship; moreover, we only studied a limited number of patients. Secondly, we did not perform any electrodiagnostic testing. Finally, we did not collect any data about the nerve pathology or the activity of the main antioxidant enzymes (GPX, SOD, and CAT) in these subjects.

## Conclusion

Altered SWM test results in diabetic patients are associated with an alteration in the glutathione system and an increase in MDA, both markers of OS. This finding may be important for understanding how OS affects DSPN in diabetic patients. However, further prospective and interventional studies will be necessary to evaluate the impact of OS in the pathogenesis of peripheral neuropathy in diabetic patients.

## Abbreviations

OS: oxidative stress
SWM: Semmes–Weinstein monofilament
T2DM: type 2 diabetes mellitus
GSH: reduced glutathione
GSSG: oxidized glutathione
MDA: malondialdehyde
DSPN: distal symmetric polyneuropathy
ROS: reactive oxygen species
AGE: advanced glycosylation end products
HbA1c: glycosylated hemoglobin
BMI: body mass index
PVD: peripheral vascular disease
ABI: ankle brachial index
HPLC: high-performance liquid chromatography assay
MDRD: modification of diet in renal disease
CMCs: circulating mononuclear cells
SPSS: statistical Package for Social Sciences
SD: standard deviation
SOD: superoxide dismutase
CAT: catalase
GPx: glutathione peroxidase
NDS: neuropathy disability score
NYHA: New York Heart Association
NSS: neuropathy sensitivity score
GFR: glomerular filtration rate
